# Unraveling the Therapeutic Mechanism of *Saussurea involucrata* against Rheumatoid Arthritis: A Network Pharmacology and Molecular Modeling-Based Investigation

**DOI:** 10.3390/nu15194294

**Published:** 2023-10-09

**Authors:** Jinghua Chen, Xiaoke Wu, Ruitao Yu

**Affiliations:** 1 Qinghai Provincial Key Laboratory of Tibetan Medicine Research, Northwest Institute of Plateau Biology, Chinese Academy of Sciences, Xining 810001, China; chenjinghua@nwipb.cas.cn (J.C.);; 2 University of Chinese Academy of Sciences, Beijing 100049, China; 3 Key Laboratory of Tibetan Medicine Research, Chinese Academy of Sciences, Xining 810001, China

**Keywords:** *Saussurea involucrata*, rheumatoid arthritis, network pharmacology, molecular docking, molecular dynamic simulation

## Abstract

Rheumatoid arthritis (RA) is a chronic autoimmune disease with a global prevalence of approximately 0.46%, causing significant impairments in patients’ quality of life and an economic burden. *Saussurea involucrata* (SI) has long been used in traditional medicine to treat RA, but its underlying mechanism remains unclear. This study utilized network pharmacology and molecular docking to explore the potential pharmacological effects of bioactive compounds in SI on RA. A total of 27 active compounds were identified, along with 665 corresponding targets. Additionally, 593 disease-related targets were obtained from multiple databases, with 119 common targets shared with SI. The high-ranking targets mainly belong to the MAPK family and NF-κB pathway, including MAPK14, MAPK1, RELA, TNF, and MAPK8, all of which are associated with inflammation and joint destruction in RA. Gene Ontology (GO) and Kyoto Encyclopedia of Genes and Genomes (KEGG) analysis revealed significant pathways related to IL-17 signaling, Th17 cell differentiation, and osteoclast differentiation. Molecular docking and dynamic simulations demonstrated strong interactions between several flavonoids and RA-related targets. Xuelianlactone, Involucratin, and Flazin exhibit outstanding binding efficacy with targets such as MAPK1, MAPK8, and TNF. These findings provide valuable insights into the therapeutic potential of SI for RA and offer directions for further drug development.

## 1. Introduction

Rheumatoid arthritis (RA), a chronic autoimmune disease, exhibited a global prevalence of 0.46% between 1980 and 2018, with significant regional and country-specific variations [1]. In mainland China, where the prevalence of RA was 0.42%, similar to the world average [2], the number of RA patients reached 5 million, with an average onset age of 45 years. Among all patients, 82% experienced moderate to severe disease, and patients often have complications [3], highlighting an ongoing and concerning situation. With a female-to-male ratio of about 4:1 in China [4], RA is more prevalent among women, and this overrepresentation is likely influenced by genetic (X-linked) factors and hormonal aspects, even though the exact reasons remain unclear [5]. The occurrence of RA is influenced by a combination of genetic and environmental factors, including smoking, infection, alcohol intake, gender, and age [6,7,8]. RA patients may suffer from various symptoms, including joint destruction, deformity, disability, and even death. In some cases, organs or systems such as the heart, kidneys, lungs, eyes, skin, digestive system, and nervous system may also be affected, leading to the development of various syndromes [9]. RA causes physical and emotional pain to patients and shortens their life expectancy. Additionally, the teratogenic and disabling effects of RA contribute to a loss of labor capacity within the population, resulting in substantial economic losses [10].

There is no cure for RA at present, but there are some pharmacological and non-pharmacological treatments that can relieve symptoms, delay disease progression, and improve quality of life [11]. The main purpose of drug treatment is to reduce inflammation, suppress excessive immune system response, and reduce joint damage. The currently used drugs include non-steroidal anti-inflammatory drugs (NSAIDs), disease-modifying anti-rheumatic drugs (DMARDs), glucocorticoids (GC), TNF inhibitors, and polymer colloids [12,13]. These drugs may cause gastrointestinal side effects such as nausea, vomiting, or abdominal pain, and adverse reactions like stomatitis or mouth sores. Moreover, they can lead to liver toxicity (hepatic dysfunction), hematological disorders, bone density reduction, insomnia, and depression. In more severe cases, patients may experience infections, cardiovascular events, cancer, or even death [14,15]. Therefore, the development of alternative drugs for RA treatment is necessary. Traditional Chinese Medicine and other ethnic medicines have a long history of clinical application and are considered valuable sources for natural drug development. These medicines offer higher safety profiles and have recently garnered significant attention in the field of RA drug research.

*Saussurea*, a medicinal plant, was first documented in the ancient Tibetan book Sman-dpyad Zla-ba’i-rgyal-po [16] in China. Among its species, *Saussurea involucrata* (SI), grown in Xinjiang, China, is particularly noteworthy and widely used in Uyghur Medicine, known as “Tage leylishi” [17]. According to the first part of the Chinese Pharmacopoeia (2020 edition), this herb is utilized in both Traditional Chinese Medicine and Uyghur Medicine to address conditions such as RA, joint pain, irregular menstruation, and excessive leucorrhea [18]. According to the current literature, SI is found to contain a variety of compounds, including phenolic acids, flavonoids, lignans, phenylpropanoids, steroids, coumarins, sesquiterpenes, ceramides, glycosides, and polysaccharides [19,20]. Ongoing studies primarily concentrate on exploring the potential therapeutic effects of its flavonoids [21,22].

In China, *Saussurea* has found extensive clinical use in the treatment of RA, available in various dosage forms such as injections, capsules, and more [23]. However, limited pharmacological studies have been conducted, with the majority of existing research focused on clinical efficacy verification. This study aims to employ network pharmacology methods to analyze the mechanism of SI in treating RA and verify the binding between its bioactive components and target proteins through molecular modeling. The findings will serve as a basis for subsequent drug development endeavors.

## 2. Materials and Methods

### 2.1. Bioactive Components of SI and Prediction of Target Genes

To ensure a comprehensive collection of the bioactive components of SI, this study conducted searches across multiple databases. The databases used included the Traditional Chinese Medicine Systems Pharmacology Database (TCMSP, tcmspw.com/tcmsp.php, accessed on 27 February 2023) [24], the Chinese Medicine and Bioactive components Database of the Shanghai Institute of Organic Chemistry (organchem.csdb.cn/scdb/default.htm, accessed on 5 April 2023) [25], and SymMap (www.symmap.org, accessed on 12 April 2023) [26]. The keyword “*Saussurea involucrata*” was employed for these searches, yielding relevant results. Furthermore, additional active ingredients were supplemented by searching the literature in the China National Knowledge Infrastructure(CNKI) [19,27,28].

The obtained components were screened based on parameters such as oral bioavailability and drug-likeness to ensure their potential for metabolism within the human body. For the query results from the SymMap database, components with an oral bioavailability (OB) threshold of ≥30% were selected [26]. These selected components, along with the components obtained from literature searches, were used to query the PubChem database (pubchem.ncbi.nlm.nih.gov, accessed on 12 April 2023) using the pubchempy Python toolkit, acquiring the Simplified Molecular Input Line Entry System (SMILES) notations. These notations were then imported into the SwissADME database (www.swissadme.ch, accessed on 16 April 2023) and subjected to screening based on criteria such as a gastrointestinal absorption (GI) rating of “High” and drug-likeness having two or more items marked as “Yes” [29]. Subsequently, the screened components were combined with the components directly obtained from the TCMSP database and underwent a further screening within TCMSP according to the standards of an oral bioavailability (OB) threshold of ≥30%, a drug-likeness (DL) threshold of ≥0.18, and a Caco-2 permeability threshold of ≥−0.4 [30,31]. Finally, all the screened components were compiled, and any duplicate entries were removed.

For the components queried from the TCMSP, their targets were also obtained from this database, and their gene names were queried from the Uniprot database (www.uniprot.org, accessed on 27 February 2023). For other components, their SMILES were input into SwissTargetPrediction (swisstargetprediction.ch, accessed on 16 April 2023) with the species attribute set to “Homo sapiens” for target prediction, and targets with a probability greater than 0 were collected [32]. Finally, all component targets were summarized and deduplicated.

### 2.2. Target Genes of RA

Disease targets were collected through searches across multiple databases. The DisGeNet database (www.disgenet.org, accessed on 6 April 2023) was queried using the keyword “rheumatoid arthritis”, and disease-related target genes were obtained by applying a screening criterion of Score ≥ 0.3 [33]. Similarly, the GeneCards database (www.genecards.org, accessed on 27 February 2023) was queried, and data with a Relevance score > 10 were selected [34]. Furthermore, the OMIM database (Online Mendelian Inheritance in Man, www.omim.org/search/advanced/geneMap, accessed on 13 April 2023) and the TTD database (Therapeutic Target Database, db.idrblab.net/ttd, accessed on 14 April 2023) were employed to query targets associated with RA [35]. Subsequently, all the retrieved results were compiled, summarized, and deduplicated to create the disease target library.

### 2.3. Protein–Protein Interaction (PPI) Network Analysis

Components exert their effects on the disease by targeting specific genes. The target genes for both the components and the disease were inputted into the Bioinformatics website (www.bioinformatics.com.cn, accessed on 17 May 2023) to generate a Venn diagram [36], obtaining the intersection of targets.

In order to elucidate the relationships among target proteins and further refine key targets, the intersecting target genes were inputted into the STRING database (string-db.org, accessed on 17 May 2023) with a confidence level set at >0.9, and the species selected as H. sapiens (human) [37]. Based on this, the PPI network was constructed. The results were then imported into Cytoscape 3.9.1 software, of which, the CytoNCA plugin was utilized to calculate various centrality metrics for each node, including betweenness centrality (BC), closeness centrality (CC), degree centrality (DC), eigenvector centrality (EC), local average connectivity-based method centrality (LAC), and network centrality (NC) [38]. The median values of each centrality metric were employed as the screening criteria, and target proteins surpassing or equaling these median values across all indicators were identified as key targets for this study.

### 2.4. Gene Ontology and Pathway Analysis

The key targets were uploaded to the Bioinformatics website for Gene Ontology (GO) and Kyoto Encyclopedia of Genes and Genomes (KEGG) analysis. The results were subjected to screening based on the criterion of *p.adjust* < 0.05 [39], and subsequently plotted using the Count, *p.adjust*, and GeneRatio values. As for the GO analysis results, they were sorted and visualized separately for the cellular component (CC), molecular function (MF), and biological process (BP) categories.

### 2.5. Network Visualization

Information such as component–target, disease–target, drug–component, pathway–target, function–target, etc., were summarized and imported into Cytoscape for network visualization, to construct their relationship network. Then, network analysis was performed using the network analysis function [37], and according to the obtained degree, 10 components and 10 targets with a higher ranking were selected as core components and core targets.

### 2.6. Molecular Docking

The core targets were searched in the RCSB PDB database (RCSB Protein Data Bank, www.rcsb.org, accessed on 18 May 2023) to retrieve structures with species as H. sapiens and a resolution of less than or equal to 2.5 Å. The structures were selected based on parameters such as Gene Name, Resolution (Å), ligand, R-Value, pH, Experimental Method, Publication Year, etc. [40]. Priority was given to structures containing similar ligands, with particular attention paid to structures with higher resolution, pH conditions closely resembling the human body environment, and more recent publication years. For each target, one structure was chosen and downloaded.

The downloaded protein structure was refined using Swiss-PdbViewer 4.1.0 [41], remove water and ligands with Pymol, and then imported into AutoDockTools for receptor preparation [42], which involved operations such as hydrogen addition and charge assignment. The structures of the core components were obtained from the PubChem database, and initial conformations for each compound were established by energy minimization using the MM2 force field in Chem3D 14.0 software [42]. After converting these files into appropriate formats, they were imported into AutoDockTools for ligand preparation.

To determine the position and size of the Grid Box, priority was given to the original ligand positions within the structures, taking into account the target position descriptions from the Uniprot database. In the case of structures without their own ligands, the Deepsite tool (www.playmolecule.com/deepsite, accessed on 28 May 2023) [43] on the PlayMolecule website was utilized for docking pocket prediction. The position and size of the grid box were determined based on the pocket scores and the 3D structure observations. Finally, AutoDock Vina 1.1.2 [42] was employed to perform molecular docking on a total of 100 receptor–ligand combinations. Each combination yielded 10 conformations along with binding energy results.

### 2.7. Molecular Dynamics (MD) Simulation

The well-combined target protein–ligand complex was selected from the molecular docking results, and MD simulations were performed using Gromacs 2023.2. For protein molecules, Charmm36 force field and TIP3P water model were chosen for processing [44]. Ligand conformations with good docking affinity were imported into Avogadro 1.2.0 to add hydrogens and format conversion. Then, the Sobtop tool (Tian Lu, Sobtop, Version 1.0 (dev3.1), http://sobereva.com/soft/Sobtop, accessed on 4 September 2023) was used to generate topology files with the GAFF force field. The receptor–ligand complex was subjected to solvation, ion balance and energy minimization. In order to simulate the human body environment, the ion concentration was set at 0.145 M when adding ions, and the MD simulation of the system was carried out at a temperature of 310 K and a pressure of 1 bar for 50 ns. The cut-off value of the non-bonded interaction was set to 1.2 nm, and the long-distance electrostatic interaction was calculated [45]. Energy calculations were carried out on complexes that exhibit favorable results in MD simulations using the gmx_mmpbsa.py [46] and gmx_mmpbsa script of gmxtool (Jicun Li, gmx_mmpbsa.bsh, DOI 10.5281/zenodo.6408973, accessed 15 September 2023).

## 3. Results

### 3.1. Bioactive Components and Key Targets

**Figure 1:**
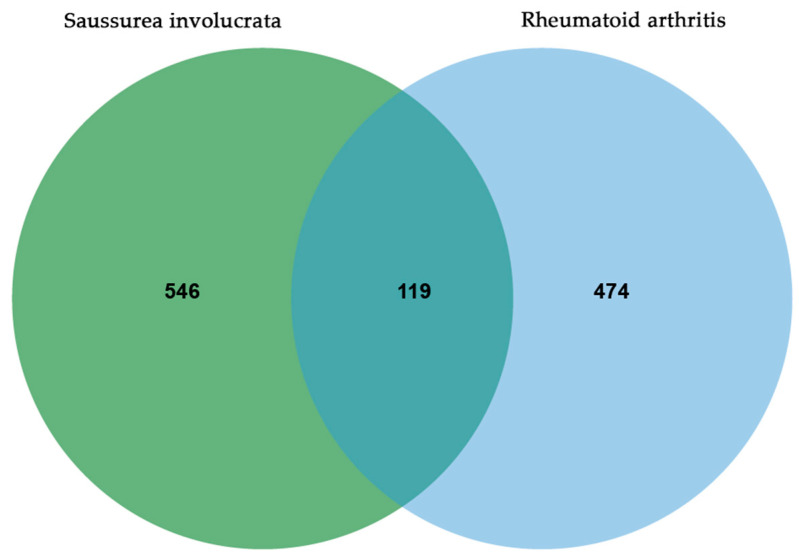
Common targets of SI and RA.

After the above component-mining, a total of 27 components of SI were obtained (Table 1), corresponding to 665 non-redundant targets. For disease targets, a total of 593 disease-related targets were obtained from four databases. By analyzing the Venn diagram, 119 intersection targets were identified, representing the overlapping targets between the component targets and the disease targets (Figure 1).

### 3.2. PPI Network Analysis

**Figure 2:**
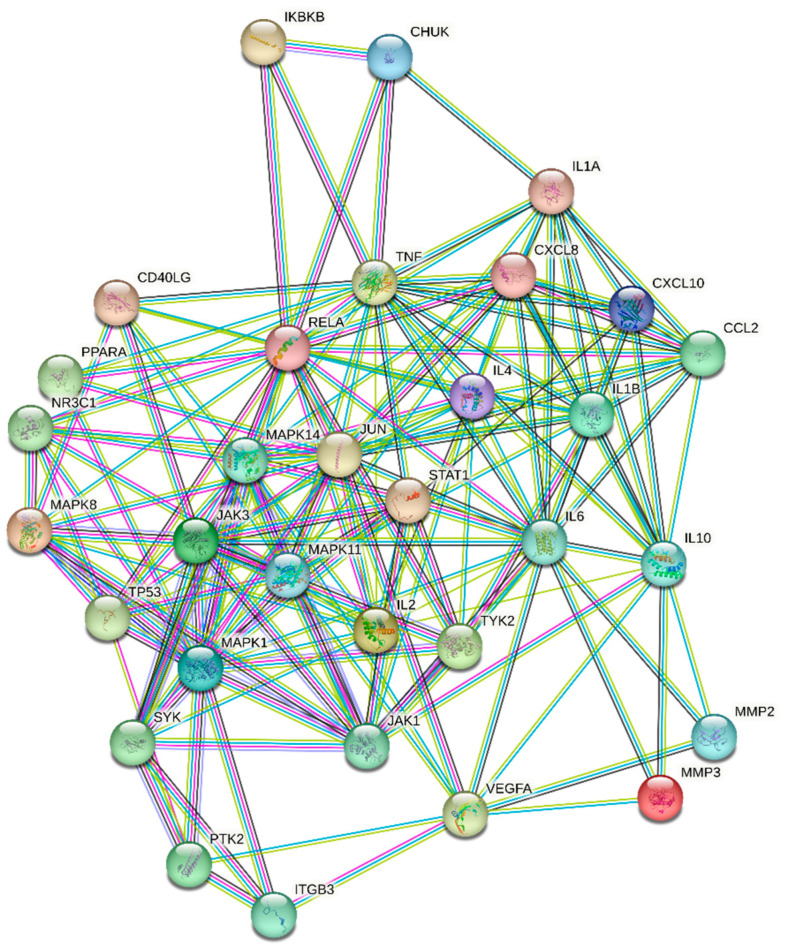
Protein–Protein Interaction Network.

Figure 2 presents the results of PPI analysis, revealing the interactions between target proteins. The significance of a target protein is determined by the number of interactions it has with other proteins. Based on the network analysis conducted in Cytoscape, a total of 33 key targets were identified (Table 2). These key targets play crucial roles in the network and are considered important in this study.

### 3.3. KEGG Pathway Enrichment Analysis

**Figure 3:**
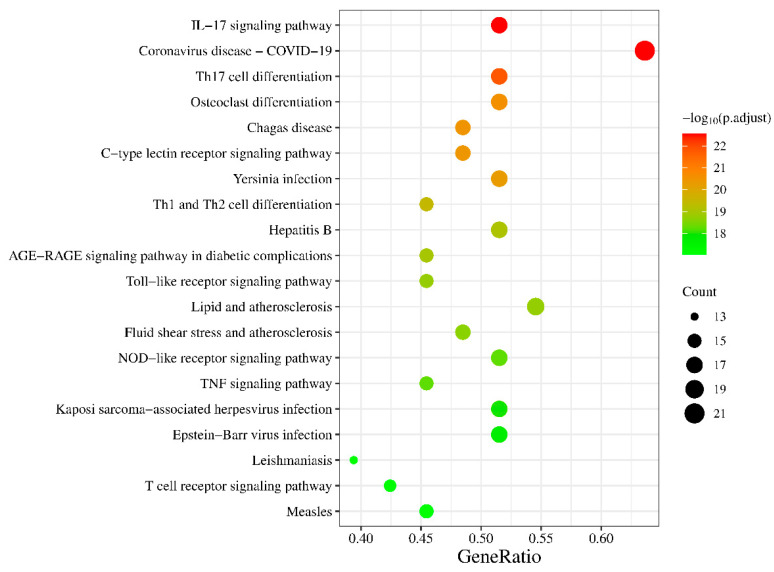
KEGG pathway enrichment analysis.

The key targets were input into the Bioinformatics website and, after screening, a total of 131 pathways with *p.adjust* < 0.05 were obtained. Among these pathways, the top 20 pathways with the smallest *p.adjust* values were selected and presented in Figure 3. The color in the plot transitions from green to red, indicating an increasing level of significance. Furthermore, the size of each circle corresponds to the number of genes enriched within the respective pathway. Larger circles signify a higher gene enrichment count.

### 3.4. GO Enrichment Analysis

**Figure 4:**
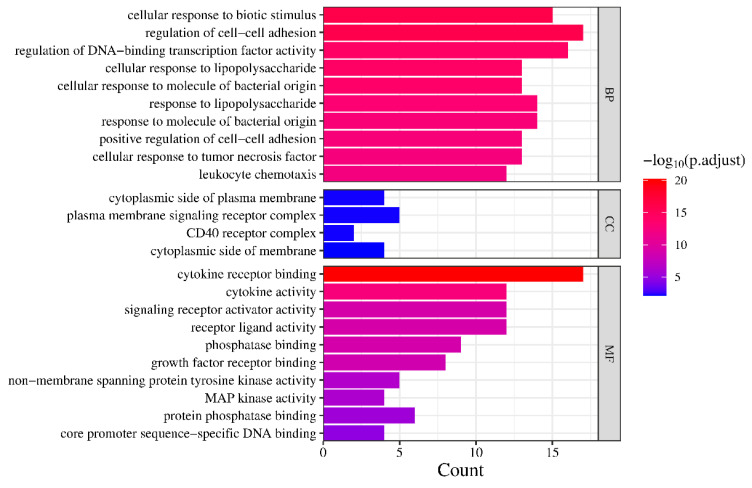
GO enrichment analysis.

Based on the output results from the Bioinformatics website, the findings were categorized into three groups, as shown in Figure 4. In both the MF and BP analyses, multiple results met the criteria of *p.adjust* < 0.05. The top 10 results with the smallest *p.adjust* values were selected for plotting. As for the CC analysis, only four results met the requirements, and all of them were included in the plot. The plot exhibits a color gradient shifting from blue to red, indicating an increasing level of significance. Additionally, the length of each bar corresponds to the number of genes enriched within the specific category, with longer bars signifying a higher gene enrichment count.

### 3.5. Network Constructions and Analysis

**Figure 5:**
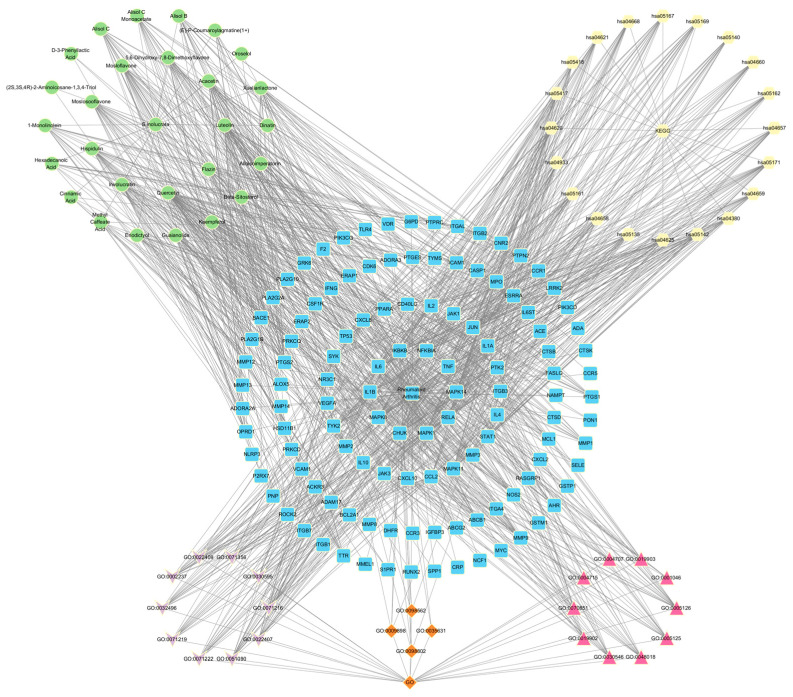
Network Analysis.

Information such as bioactive components, key targets, related pathways, and the functions obtained above was formatted in a network diagram using Cytoscape software (Figure 5). In the diagram, green circles represent the bioactive components present in SI, which intersect with RA-related targets. The intersection results are depicted by blue squares. Among them, the inner two circles represent the 33 key targets identified through network analysis, while the innermost circle represents the 10 core targets selected for molecular docking. Yellow hexagons represent the top 20 pathways obtained from the key target analysis. At the bottom of the diagram, three distinct groups illustrate the results of the GO analysis. Purple, orange, and red represent the BP, CC, and MF analysis results, respectively. This comprehensive analysis unveils the intrinsic correlation between the bioactive components of SI and their potential in treating RA.

### 3.6. Molecular Docking

**Figure 6:**
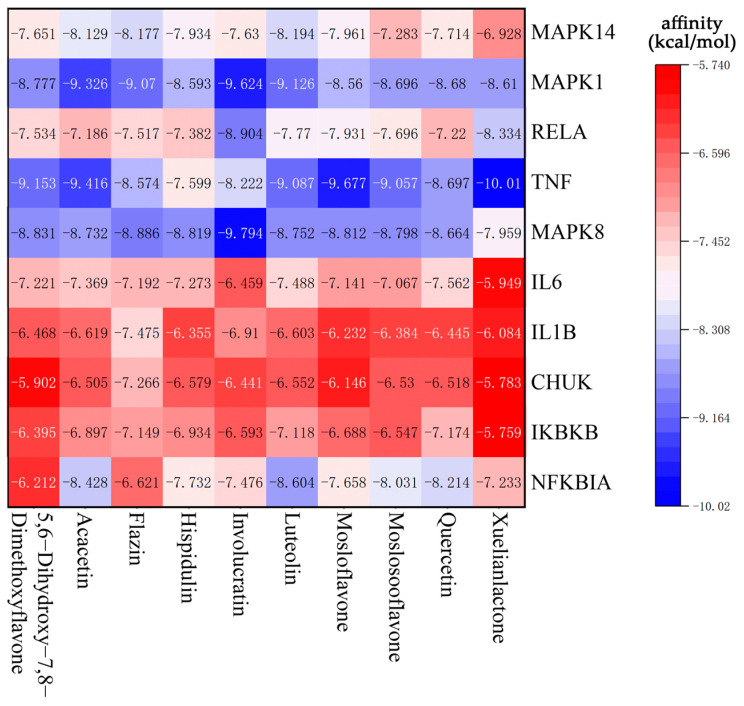
Heat map of lowest binding energy in molecular docking.

According to the network analysis results of Cytoscape, 10 compounds with the highest degree values, Quercetin, Luteolin, Acacetin, Xuelianlactone, Moslosooflavone, Hispidulin, Mosloflavone, Involucratin, 5,7-Dihydroxy-6,8-Dimethoxyflavone and Flazin were selected as molecular docking ligands (core components). Ten targets with the highest degree values, MAPK14, MAPK1, RELA, TNF, MAPK8, IL6, IL1B, CHUK, IKBKB and NFKBIA, were selected as the core targets for molecular docking. Nine protein structures were selected as the receptors, with CHUK and IKBKB targets located in different segments of the same protein structure (Table 3). The results of the molecular docking were visualized in the form of a heat map (Figure 6), where the color gradient shifting from red to blue indicates a decrease in affinity. Lower binding energy signifies a better binding activity between the ligand and receptor protein. In general, affinity values > −5 kcal/mol indicate no predicted binding, affinity < −5 kcal/mol suggests moderate predicted binding, and affinity < −7 kcal/mol suggests strong predicted binding [47]. In this section, all the selected ligands and receptors demonstrated excellent binding activity, as the lowest binding energy with the best ligand for each receptor was less than −7 kcal/mol. Notably, Xuelianlactone demonstrates the lowest affinity at −10.01 kcal/mol. In addition, the docking results of MAPK1, MAPK8, and TNF showed significantly lower binding affinities compared to the other targets (*p* < 0.05). However, the binding effect of IL6, ILB, CHUK, and IKBKB to each ligand is not outstanding.

**Figure 7:**
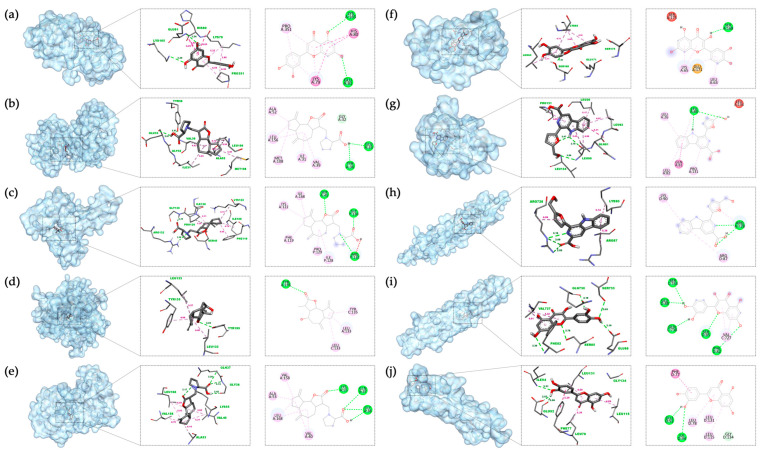
Molecular docking results in each target with the bioactive compound of SI: (**a**) Luteolin and MAPK14; (**b**) Involucratin and MAPK1; (**c**) Involucratin and RELA; (**d**) Xuelianlactone and TNF; (**e**) Involucratin and MAPK8; (**f**) Quercetin and IL6; (**g**) Flazin and IL1B; (**h**) Flazin and CHUK; (**i**) Quercetin and IKBKB; (**j**) Luteolin and NFKBIA. The images depict the binding positions of ligands within the protein, along with the surrounding residues, showcasing the interactions between the ligands and the residues. In this figure, pink, magenta, and purple dashed lines represent hydrophobic interactions, while the green dashed lines represent hydrogen bonds.

For each target protein, the ligand with the lowest affinity was selected from the molecular docking results. Visualization was achieved using Biovia Discovery Studio 2021 software, and the docking results are presented in Table 4 and Figure 7.

### 3.7. Molecular Dynamics Simulation

**Figure 8:**
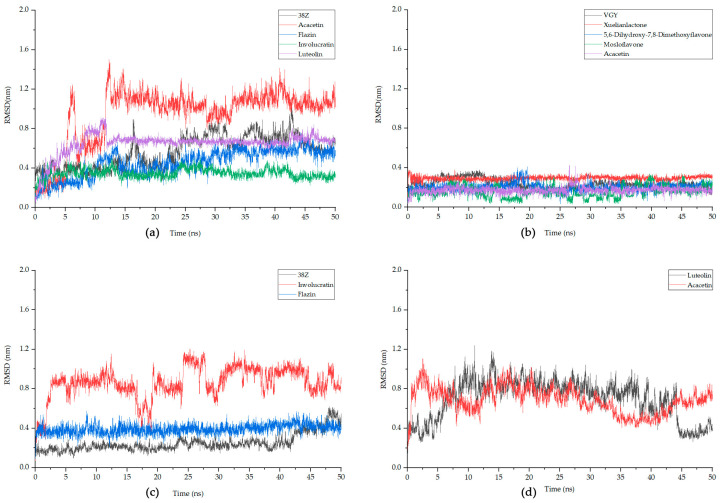
The fluctuation plot of the target protein–ligand complexes RMSD. (**a**) MAPK1, (**b**) TNF, (**c**) MAPK8, (**d**) NFKIBA. 38Z is the original ligand for MAPK1 and MAPK8, while VGY is for TNF, and NFKIBA does not have an original ligand.

Based on the degree values obtained in network analysis and molecular docking results, the following study primarily focuses on the MAPK1 and TNF. For both targets, the top four compounds with the best docking results, as well as the original ligands (38Z and VGY) of the target proteins, were selected for MD simulations. In addition, for MAPK8, two compounds and the original ligand (38Z) were chosen for simulations. Considering the certainty of the target position, the simulation was conducted with NFKIBA as the representative target for the NF-κB pathway, focusing on the top two best-performing compounds based on the docking results. Table 5 and Figure 8 present the Root Mean Square Deviation (RMSD) values for each of these simulation combinations.

In MD simulations, RMSD measures the average deviation or fluctuations in atomic positions in a molecular system from their initial positions, providing insight into the stability of a target protein–ligand complex. Generally, it is considered that once the RMSD value stabilizes and the fluctuation range is less than 0.2 nm, the system can be deemed stable [48]. It can be observed that, for MAPK1, Involucratin and Luteolin reach equilibrium after about 13 ns, with smaller fluctuations than the protein’s original ligand 38Z. The stabilized RMSD value for Involucratin is less than 0.4 nm, and both compounds exhibit fluctuations below 0.2 nm after stabilization. For TNF, the RMSD values for all analyzed compounds remain below 0.4 nm, with fluctuations below 0.2 nm for all except Mosloflavone. Among them, Xuelianlactone and Acacetin demonstrate the highest stability. Regarding MAPK8, the original ligand 38Z exhibits a low RMSD value, but shows significant fluctuations towards the end of the simulation. On the other hand, Flazin maintains an RMSD of 0.39, with fluctuations also within the 0.2 nm range. As for NFKIBA, both selected ligand Luteolin and Acacetin display higher overall fluctuations. In the last 5 ns, their RMSDs tend to stabilize, with fluctuations smaller than 0.2 nm. However, compared to other complexes, their stability remains poor, and thus, they cannot be considered to have achieved stable binding. Based on this, further analysis will be conducted of the following compounds: Involucratin and Luteolin for MAPK1, Xuelianlactone and Acacetin for TNF, and Flazin for MAPK8.

**Figure 9:**
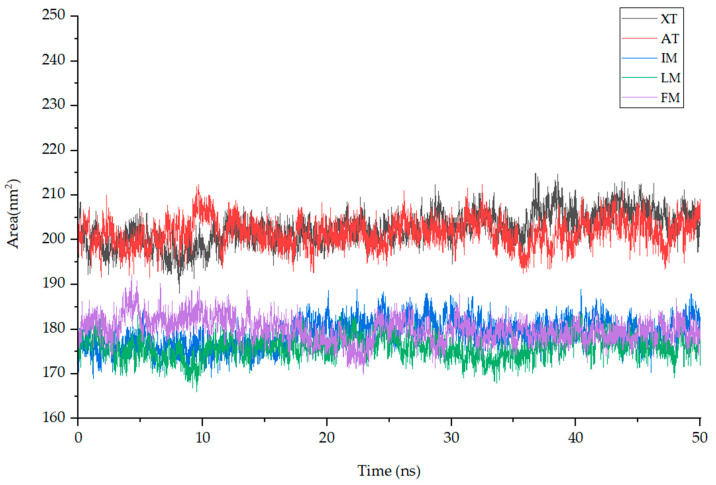
The fluctuation plot of the target protein–ligand complexes SASA. **Note:** XT: Xuelianlactone-TNF(7JRA), AT: Acacetin-TNF(7JRA), IM: Involucratin-MAPK1(4QTA), LM: Luteolin-MAPK1(4QTA), FM: Flazin-MAPK8(4QTD).

Solvent-Accessible Surface Area (SASA) quantifies the surface area of a molecule that is accessible to solvent molecules and can indicate changes in a molecule’s conformation or exposure during simulation, aiding in the assessment of binding interactions in a target protein–ligand complex. The average SASA values for complexes XT (black), AT (red), IM (blue), LM (green), and FM (purple) during MD simulation are as follows: 202.49 ± 3.72, 201.65 ± 3.17, 179.03 ± 3.25, 175.97 ± 2.59, and 179.9 ± 2.9 nm^2^. Throughout the entire simulation process, the SASA values of these compounds show small fluctuations, indicating their stable interaction with the protein complexes (Figure 9).

**Figure 10:**
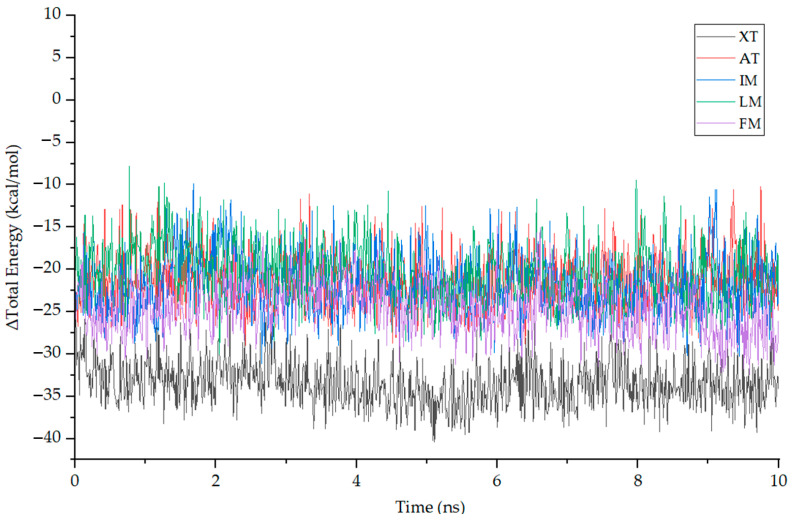
The fluctuation plot of the target protein–ligand complexes ΔH.

Molecular Mechanics–Poisson Boltzmann Surface Area (MMPBSA) is a method used to estimate the binding energy of a target protein–ligand complex by post-processing the MD trajectory. MMPBSA can characterize the strength and stability of the protein–ligand interaction by calculating the difference between the free energy of the bound state and the free energy of the unbound state. For the five complexes mentioned above, we selected segments with stable RMSD values and extracted 10 ns trajectories for MMPBSA energy analysis. The total binding energy (ΔG) is the sum of the change in enthalpy (ΔH) and the change in entropy (–TΔS) [49]. Using gmx_mmpbsa.py with a time interval of 10 ps, the total energy difference (ΔG_total_) between the ligand, receptor and their respective complexes in the stable phase of MD simulation was calculated, as shown in Figure 10. It can be seen from the figure that the ΔG_total_ curves of each compound have small fluctuations, and the average is below −10 kcal/mol. Furthermore, by utilizing the gmx_mmpbsa.bsh script, the entropy change in each complex is calculated at a time interval of 1000 ps, allowing for the calculation of the total binding energy (ΔG). Table 6 presents the contributions of the Van der Waals force (VDW), coulomb interaction energy (COU), molecular mechanics (MM), polar solvation energy (PB) and nonpolar solvation energy (SA). The ΔH calculated by gmx_mmpbsa.bsh closely aligns with the ΔG_total_ computed using gmx_mmpbsa.py. The results from both methods exhibit a close resemblance, which enhances the credibility of the computed free energy values. It can be observed from the figure and the table that ΔG and ΔH for XT are significantly lower than that of the other ligands, consistent with the results observed in molecular docking.

**Figure 11:**
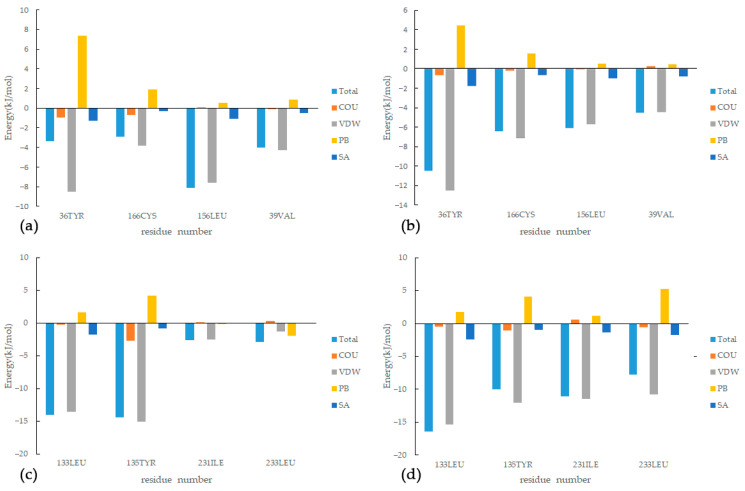
Energy decomposition of key residues: (**a**) IM, (**b**) LM, (**c**) XT, (**d**) AT.

This study investigated the binding free energy and residue contributions of active components of SI to the RA target using methods such as MD simulations and MMPBSA energy calculations. As shown in Figure 11, among the four complexes, Van der Waals forces have the most significant impact in promoting protein–ligand binding, while nonpolar solvation energy has a relatively weaker influence on binding affinity. On the other hand, polar solvation energy strongly impedes the formation of the complexes. Specifically, for the MAPK1 target, we compared the results of MD simulations for the IM and LM complexes and identified four residues (36, 39, 156, and 166) that played important roles in both complexes. In the LM complex, the sum of the ΔH values for these four residues accounted for 42.9% of the total ΔH. The maximum resistance to binding was observed from residues such as 54, 151, 153, and 167, but it was insufficient to offset the binding energy. For the TNF target, in both the XT and AT complexes, four residues (133, 135, 231, and 233) made significant contributions to the binding free energy. In the AT complex, the sum of the ΔH values for these four residues accounted for 43.0% of the total ΔH, while the maximum resistance to binding came from residues 174 and 195, although their impact was relatively small, with residue 174 contributing only 0.687 kJ/mol to ΔH. Compared to complexes IM and LM, complexes AT and XT exhibit higher binding energy contributions from residues and a lower binding resistance. This observation aligns with what is reflected in the RMSD curves.

The analysis of residue interactions with different ligands targeting the same receptor reveals crucial regions that facilitate complex binding. In future drug design efforts, it is essential to pay attention to the contributions of these residues. This can be achieved by introducing functional groups or optimizing their molecular spatial structures, aiming to retain residues with strong binding forces and reduce the impact of residues with significant resistance [50].

## 4. Discussion

In this study, we employed network pharmacology to analyze SI, resulting in the identification of 27 bioactive components and 119 targets that intersect with RA. Among these targets, 33 were identified as key targets. Additionally, we conducted KEGG and GO analyses to investigate the pathways and biological processes involved. The findings suggest a potential correlation between the components present in SI and the treatment of RA.

Among the core targets, ERK (MAPK1), JNK (MAPK8), and p38 MAPK (MAPK14) are well-defined families of MAPKs, activated by phosphorylation, and play significant roles in the inflammatory and destructive mechanisms observed in rheumatoid arthritis, including regulating pro-inflammatory cytokine production and mediating downstream signaling from IL-1, IL-17, and TNF-α receptors. These properties make them attractive therapeutic targets for rheumatic diseases [51,52]. RELA, NFKBIA, CHUK, and IKBKB are associated with the NF-κB pathway. CHUK and IKBKB serve as different catalytic subunits of the IκB kinase (IKK) complex [53,54]. Highly activated NF-κB induces the production of pro-inflammatory cytokines, including TNF-α, IL-1β, and IL-6, accelerating RA progression. The upregulation of these cytokines also triggers positive feedback, regulating NF-κB activation, and forming a vicious cycle that worsens RA development [52]. Among these targets, MAPK targets exhibit a higher degree of centrality in network analysis. According to molecular docking results, both MAPK1 and MAPK8 demonstrate prominent binding efficiency with all core components. Therefore, MAPK targets play a crucial role in the process of treating RA with IS. Regarding NF-κB-related targets, their network analysis shows lower degree centrality rankings, and taking NFKIBA as an example, the results of molecular dynamics simulations fail to confirm its stable binding. Therefore, it can be inferred that the active components of SI have a weaker effect on these targets.

From a pathway perspective, the pathways exhibiting high gene enrichment and confidence levels include IL-17, TNF, TLR (Toll-like receptor), NLR (NOD-like receptor), and CLR (C-type lectin receptor) signaling pathways, as well as Th17, Th1, and Th2 cell differentiation pathways, among which the IL-17 and Th17 pathways are the most confident. Interactions between RA, Fibroblast-Like Synoviocytes (FLSs), and Th17 cells further contribute to tumorous FLS growth and joint pannus formation [55]. Previous research has identified IL-17 as a potential therapeutic target for RA, leading to several trials being conducted [56,57]. There is also considerable confidence in the osteoclast (OC) differentiation pathway, which plays a key role in the pathological bone destruction observed in RA. Pro-inflammatory cytokines, including TNF, IL-1 and IL-17, induce OC maturation and differentiation through different signaling pathways such as NF-κB [58]. This highlights the dual therapeutic role of SI, not only targeting the inflammatory symptoms of RA but also reducing bone destruction.

The GO analysis primarily centers on responses to biotic stimuli, specifically lipopolysaccharides and other molecules of bacterial origin, known to trigger immune and inflammatory responses. Previous research has indicated that bacterial infection plays a significant role in contributing to RA pathogenesis [59]. The pathways showing the highest enrichment, such as Coronavirus disease, Chagas disease, and Yersinia infection, further reinforce this notion, as they are involved in immune responses to viruses, parasites, and bacteria. In terms of molecular function analysis, the active components primarily influence functions related to binding activities, such as cytokine receptor binding, growth factor receptor binding, and phosphatase binding, while also affecting cytokine activity and receptor ligand activity. This suggests that these components may modulate processes like phosphorylation within the pathways by binding to specific targets, thereby inhibiting excessive immune responses and achieving therapeutic effects. Additionally, in the biological process analysis, certain processes are associated with the regulation of cell–cell adhesion. Adhesion molecules are important regulators of leukocyte recruitment into the synovial tissue [60], while Leukocytes, which are aberrantly recruited and activated, are characteristic features of rheumatoid arthritis (RA), as they migrate out of the blood, cross the endothelium, and infiltrate inflamed tissues, triggering a cascade of pathological processes, particularly in the early stages of the disease [61].

Based on the above analysis results, components Luteolin, Involucratin, Xuelianlactone, Flazin, Quercetin, and Acacetin, all belonging to the flavonoid class, exhibit favorable binding effects with the targets. Quercetin exerted a bone-protective effect by reducing MMPs, RANKL production, and osteoclast formation via the MAPKs and NF-κB pathways [62]. Current animal model studies have demonstrated the effectiveness of quercetin in treating RA [63]. Some studies suggest that Luteolin can inhibit the proliferation and affect the function of stimulated rat synovial fibroblasts [64]. Furthermore, it has the ability to suppress the IL-1β-induced production of cytokines and MMPs, which are crucial in tissue degradation in rheumatoid synovium, through the activation of p38 MAPK, JNK, NF-κB, and AP-1 [65]. Acacetin inhibits p38 and JNK phosphorylation and reduces MMP-1, MMP-3, and MMP-13 expression in IL-1β-induced FLSs, suggesting that Acacetin has antiarthritic effects in FLSs [66]. In addition, it has been shown to inhibit the activation of NF-κB by stimulation with TNF-α [67]. Moreover, this study reveals the significant roles of Involucratin, Xuelianlactone, and Flazin in the process of treating RA with SI. Among all complexes, Xuelianlactone exhibits the best binding efficiency with the TNF target, displaying the lowest binding energy in both molecular docking and MD simulations, along with good binding stability. Involucratin, on the other hand, stands out in the molecular docking results, showing the lowest binding energy among ligands binding to MAPK1, MAPK8, and RELA. Flazin also demonstrates strong binding abilities with MAPK8 in MD simulations. Considering the limited number of related studies conducted on these compounds to date, they represent potential new research avenues for the development of RA treatment drugs in the future.

## 5. Conclusions

In summary, the network pharmacology analysis suggests that the mechanism of SI in treating RA mainly involves its flavonoids, which target key proteins through MAPK and NF-κB-related pathways. By doing so, they effectively inhibit inflammation and mitigate bone destruction, leading to therapeutic effects. The binding efficiency of complexes like XT, AT, and FM has been confirmed through MD simulations. This suggests that the action of SI in treating RA is achieved through multiple components acting on various targets. However, it is essential to note that network pharmacology is primarily a data-driven and network-based research method. Therefore, these findings should be further validated and supported by experimental studies to establish their clinical relevance and potential as RA treatment options.

## Figures and Tables

**Table 1:** Bioactive components of SI.

No.	Name	Molecular Formula	Molecular Weight	PubChem CID
1	Dinatin	C_16_H_12_O_6_	300.26	5281628
2	Alloisoimperatorin	C_16_H_14_O_4_	270.28	5317436
3	Beta-Sitosterol	C_29_H_50_O	414.70	222284
4	Kaempferol	C_15_H_10_O_6_	286.24	5280863
5	Luteolin	C_15_H_10_O_6_	286.24	5280445
6	Flazin	C_17_H_12_N_2_O_4_	308.29	5377686
7	Quercetin	C_15_H_10_O_7_	302.23	5280343
8	Involucratin	C_20_H_27_NO_4_	345.40	15628146
9	Guaianolide	C_14_H_20_O_3_	236.31	-
10	Eriodictyol	C_15_H_12_O_6_	288.25	440735
11	Hispidulin	C_16_H_12_O_6_	300.26	5281628
12	Methyl Caffeate Acid	C_10_H_10_O_4_	194.18	689075
13	Cinnamic Acid	C_9_H_8_O_2_	148.16	444539
14	Hexadecanoic Acid	C_16_H_32_O_2_	256.42	985
15	1-Monolinolein	C_21_H_38_O_4_	354.50	6256628
16	(2S,3S,4R)-2-Aminoicosane-1,3,4-Triol	C_20_H_43_NO_3_	345.60	12302752
17	Moslosooflavone	C_17_H_14_O_5_	298.29	188316
18	Mosloflavone	C_17_H_14_O_5_	298.29	471722
19	5,6-Dihydroxy-7,8-Dimethoxyflavone	C_17_H_14_O_6_	314.29	821356
20	D-3-Phenyllactic Acid	C_9_H_10_O_3_	166.17	643327
21	Alisol C	C_30_H_46_O_5_	486.70	101306923
22	Alisol C Monoacetate	C_32_H_48_O_6_	528.70	14036813
23	(E)-P-Coumaroylagmatine(1+)	C_14_H_21_N_4_O_2_^+^	277.34	25245514
24	Alisol B	C_30_H_48_O_4_	472.70	15558620
25	Acacetin	C_16_H_12_O_5_	284.26	5280442
26	Oroselol	C_14_H_12_O_4_	244.24	160600
27	Xuelianlactone	C_15_H_20_O_3_	248.32	147111

**Table 2:** List of Key Targets.

No.	Gene Symbol	Gene ID	Gene Name
1	TNF	7124	Tumor necrosis factor
2	RELA	5970	Transcription factor p65
3	IL6	3569	Interleukin-6
4	JUN	3725	Proto-oncogene c-JUN
5	MAPK1	5594	Mitogen-activated protein kinase 1
6	MAPK14	1432	MAP kinase p38 alpha
7	IL10	3586	Interleukin-10
8	IL1B	3553	Interleukin-1 beta
9	CCL2	6347	C-C motif chemokine 2
10	IL4	3565	Interleukin-4
11	IL2	3558	Interleukin-2
12	STAT1	6772	Signal transducer and activator of transcription 1-alpha/beta
13	CXCL8	3576	Interleukin-8
14	IL1A	3552	Interleukin-1 alpha
15	JAK1	3716	Tyrosine-protein kinase JAK1
16	CXCL10	3627	C-X-C motif chemokine 10
17	NFKBIA	4792	NF-kappa-B inhibitor alpha
18	JAK3	3718	Tyrosine-protein kinase JAK3
19	TP53	7157	Cellular tumor antigen p53
20	VEGFA	7422	Vascular endothelial growth factor A
21	MAPK11	5600	MAP kinase p38 beta
22	TYK2	7297	Tyrosine-protein kinase TYK2
23	MAPK8	5599	Mitogen-activated protein kinase 8
24	ITGB3	3690	Integrin alpha-2/beta-3
25	NR3C1	2908	Glucocorticoid receptor
26	PPARA	5465	Peroxisome proliferator-activated receptor alpha
27	PTK2	5747	Focal adhesion kinase 1
28	CHUK	1147	Inhibitor of nuclear factor kappa-B kinase subunit alpha
29	IKBKB	3551	Inhibitor of nuclear factor kappa-B kinase subunit beta
30	MMP3	4314	Stromelysin-1
31	MMP2	4313	72 kDa type IV collagenase
32	CD40LG	959	CD40 ligand
33	SYK	6850	Tyrosine-protein kinase SYK

**Table 3:** Core components.

No.	Ingredients	Degree
1	Quercetin	60
2	Luteolin	43
3	Acacetin	33
4	Xuelianlactone	26
5	Moslosooflavone	25
6	Hispidulin	24
7	Mosloflavone	24
8	Involucratin	23
9	5,6-Dihydroxy-7,8-Dimethoxyflavone	23
10	Flazin	21

**Table 4:** Best protein–ligand complexes and their binding energies.

No.	Receptor	Degree of Receptor	Structure	Target Chain	Target Position	Ligand	Affinity (kcal/mol)
1	MAPK14	38	5WJJ	A	1–360	Luteolin	−8.194
2	MAPK1	37	4QTA	A	1–360	Involucratin	−9.624
3	RELA	35	6NV2	P	39–51	Involucratin	−8.904
4	TNF	33	7JRA	A/B/C	77–233	Xuelianlactone	−10.01
5	MAPK8	33	4QTD	A	1–363	Involucratin	−9.794
6	IL6	33	7NXZ	A	30–212	Quercetin	−7.562
7	IL1B	31	5R8Q	A	117–269	Flazin	−7.475
8	CHUK	31	3BRT	A/C	732–745	Flazin	−7.266
9	IKBKB	30	3BRT	A/C	701–730	Quercetin	−7.174
10	NFKBIA	28	1IKN	D	67–302	Luteolin	−8.604

**Table 5:** The Average RMSD values for each target protein–ligand complex.

**Target**	MAPK1				
**Compounds**	38Z	Acacetin	Flazin	Involucratin	Luteolin
**RMSD Average (nm)**	0.567 ± 0.162	0.940 ± 0.290	0.455 ± 0.139	0.346 ± 0.048	0.650 ± 0.112
**Target**	TNF				
**Compounds**	VGY	Xuelianlactone	5,6-Dihydroxy-7,8- Dimethoxyflavone	Mosloflavone	Acacetin
**RMSD Average (nm)**	0.235 ± 0.049	0.295 ± 0.022	0.205 ± 0.040	0.155 ± 0.060	0.175 ± 0.034
**Target**	MAPK8			NFKIBA	
**Compounds**	38Z	Involucratin	Flazin	Luteolin	Acacetin
**RMSD Average (nm)**	0.251 ± 0.091	0.862 ± 0.160	0.390 ± 0.049	0.698 ± 0.199	0.686 ± 0.132

**Table 6:** The ΔG of each complex and the contributions of individual energy components(kcal/mol).

Complex	Contribution							
	ΔG	−TΔS	ΔH	MM	PB	SA	COU	VDW
XT	−30.03	5.35	−35.38	−45.34	14.48	−4.53	−3.20	−42.13
AT	−23.42	1.73	−25.15	−41.16	21.43	−5.42	−5.74	−35.42
IM	−11.96	3.69	−15.65	−45.88	35.93	−5.71	−9.88	−35.99
LM	−10.99	3.74	−14.73	−43.06	33.36	−5.03	−10.11	−32.95
FM	−22.00	2.89	−24.89	−43.49	23.80	−5.20	−5.47	−38.02

Note: In this table, MM is the sum of VDW and COU, while ΔH is the sum of MM, PB, and SA.

## Data Availability

The original data are available upon reasonable request to the corresponding author.

## References

[1] Almutairi K., Nossent J., Preen D., Keen H., Inderjeeth C. (2021). The global prevalence of rheumatoid arthritis: A meta-analysis based on a systematic review. Rheumatol. Int..

[2] Zeng X., Zhu S., Tan A., Xie X. (2013). Disease burden and quality of life of rheumatoid arthritis in China: A systematic review. Chin. J. Evid. Based Med..

[3] Zeng X., Tian X., Li M. (2021). China Rheumatoid Arthritis Development Report 2020.

[4] Tian X., Wang Q., Li M., Zhao Y., Zhang Z., Huang C., Liu Y., Xu H., Chen Y., Wu L. (2021). 2018 Chinese guidelines for the diagnosis and treatment of rheumatoid arthritis. Rheumatol. Immunol. Res..

[5] van Vollenhoven R.F. (2009). Sex differences in rheumatoid arthritis: More than meets the eye. BMC Med..

[6] Goemaere S., Ackerman C., Goethals K., De Keyser F., Van der Straeten C., Verbruggen G., Mielants H., Veys E. (1990). Onset of symptoms of rheumatoid arthritis in relation to age, sex and menopausal transition. J. Rheumatol..

[7] Hitchon C.A., El-Gabalawy H.S. (2011). Infection and rheumatoid arthritis: Still an open question. Curr. Opin. Rheumatol..

[8] Jiang X., Alfredsson L. (2020). Modifiable environmental exposure and risk of rheumatoid arthritis—Current evidence from genetic studies. Arthritis Res. Ther..

[9] Wu D., Luo Y., Li T., Zhao X., Lv T., Fang G., Ou P., Li H., Luo X., Huang A. (2022). Systemic complications of rheumatoid arthritis: Focus on pathogenesis and treatment. Front. Immunol..

[10] Zhang W., Anis A.H. (2011). The economic burden of rheumatoid arthritis: Beyond health care costs. Clin. Rheumatol..

[11] Fidahic M., Kadic A.J., Radic M., Puljak L. (2017). Celecoxib for rheumatoid arthritis. Cochrane Database Syst. Rev..

[12] Yeo J., Lee Y.M., Lee J., Park D., Kim K., Kim J., Park J., Kim W.J. (2019). Nitric oxide-scavenging nanogel for treating rheumatoid arthritis. Nano Lett..

[13] Abbasi M., Mousavi M.J., Jamalzehi S., Alimohammadi R., Bezvan M.H., Mohammadi H., Aslani S. (2019). Strategies toward rheumatoid arthritis therapy; the old and the new. J. Cell. Physiol..

[14] Shea B., Swinden M.V., Ghogomu E.T., Ortiz Z., Katchamart W., Rader T., Bombardier C., Wells G.A., Tugwell P. (2013). Folic acid and folinic acid for reducing side effects in patients receiving methotrexate for rheumatoid arthritis. Cochrane Database Syst. Rev..

[15] Tu A.B., Lewis J.S. (2021). Biomaterial-based immunotherapeutic strategies for rheumatoid arthritis. Drug Deliv. Transl. Res..

[16] Junshan L., Shaoqing C. (1998). Advances in Chemical and Pharmacological Research of Saussurea. Chin. Pharm. J..

[17] Ruiping Y., Yongfeng J., Liang W. Research on Drug Resources of Saussurea. Proceedings of the International Congress of Traditional Medicine.

[18] Committee N.P. (2020). Pharmacopoeia of People’s Republic of China. Part 1.

[19] Fan W.-X., Yang W.-P., Liu H.-S. (2021). Research progress on culture technologies, chemical components, and pharmacological activities of Saussurea involucrata cells. Chin. J. Chin. Mater. Med..

[20] Chik W.-I., Zhu L., Fan L.-L., Yi T., Zhu G.-Y., Gou X.-J., Tang Y.-N., Xu J., Yeung W.-P., Zhao Z.-Z. (2015). Saussurea involucrata: A review of the botany, phytochemistry and ethnopharmacology of a rare traditional herbal medicine. J. Ethnopharmacol..

[21] Cao Z.-X., Li H.-H., Li A., Liu P.-Y., Zhao Y.-Y., Mao P.-Q., Li G.-L. (2018). Analysis of flavonoids and antitumor activity of transgenic Saussurea involucrate. Chin. J. Chin. Mater. Med..

[22] Jing L., He L., Fan P., Jia Z., Ma H. (2015). Chemical constituents with anti-hypoxia activity from Saussurea involucrata. Zhong Yao Cai.

[23] Huan W., Qiu X., Xu F. (2008). A systematic review clinical efficacy about Xuelian treatment for rheumatoid arthritis. Chin. Pract. Med..

[24] Song S., Zhou J., Li Y., Liu J., Li J., Shu P. (2022). Network pharmacology and experimental verification based research into the effect and mechanism of Aucklandiae Radix–Amomi Fructus against gastric cancer. Sci. Rep..

[25] Na Z., Fengrong Z., Huayong T., Qingqing L., Xiulan H., Zhiyong L. (2022). Benefit evaluation of Tujia medicine Tianzhusan added medicine prescription based on traditionaknowledge investigation and network pharmacology. J. Hunan Univ. Chin. Med..

[26] Ye X.W., Wang H.L., Cheng S.Q., Xia L.J., Xu X.F., Li X.R. (2022). Network Pharmacology-Based Strategy to Investigate the Pharmacologic Mechanisms of Coptidis Rhizoma for the Treatment of Alzheimer’s Disease. Front. Aging Neurosci..

[27] Yong Y., Jianguo X., Guihua L., Yongjun Z., Xincun W., Guipeng X. (2011). In situ rat intestinal absorption of two active components in Saussurea involucrate. Chin. J. Hosp. Pharm..

[28] Wu Q., Ma J., Shu Z., Ren Y., Li D.-Z., Zhang Y.-L. (2021). Potential therapeutic effect of Saussureae Involucratae Herba on breast cancer and its mechanism based on network pharmacology. Chin. J. Chin. Mater. Med..

[29] Wang D., Sun Y., Liu Q., Ye C., Zhao S., Zhang H. (2023). Ferula sinkiangensis against gastric cancer: A network pharmacology, molecular docking and cell experiment study. Transl. Cancer Res..

[30] Dong Y., Zhao Q., Wang Y. (2021). Network pharmacology-based investigation of potential targets of astragalus membranaceous-angelica sinensis compound acting on diabetic nephropathy. Sci. Rep..

[31] Choi N.-R., Jung D., Kim S.-C., Park J.-W., Choi W.-G., Kim B.-J. (2023). Analysis of Network Pharmacological Efficacy and Therapeutic Effectiveness in Animal Models for Functional Dyspepsia of Foeniculi fructus. Nutrients.

[32] Khan S.A., Lee T.K.W. (2022). Network-pharmacology-based study on active phytochemicals and molecular mechanism of cnidium monnieri in treating hepatocellular carcinoma. Int. J. Mol. Sci..

[33] Shi H., Tian S., Tian H. (2021). Network pharmacology interpretation of fuzheng–jiedu decoction against colorectal cancer. Evid. Based Complement. Alternat. Med..

[34] Hu H., Wang H., Yang X., Li Z., Zhan W., Zhu H., Zhang T. (2023). Network pharmacology analysis reveals potential targets and mechanisms of proton pump inhibitors in breast cancer with diabetes. Sci. Rep..

[35] Wang Y., Yuan Y., Wang W., He Y., Zhong H., Zhou X., Chen Y., Cai X.J., Liu L.Q. (2022). Mechanisms underlying the therapeutic effects of Qingfeiyin in treating acute lung injury based on GEO datasets, network pharmacology and molecular docking. Comput. Biol. Med..

[36] Wu Y., Liu X., Li G. (2022). Integrated bioinformatics and network pharmacology to identify the therapeutic target and molecular mechanisms of Huangqin decoction on ulcerative Colitis. Sci. Rep..

[37] Liu H., Mohammed S.A.D., Lu F., Chen P., Wang Y., Liu S. (2022). Network Pharmacology and Molecular Docking-Based Mechanism Study to Reveal Antihypertensive Effect of Gedan Jiangya Decoction. BioMed Res. Int..

[38] Bai X., Tang Y., Li Q., Chen Y., Liu D., Liu G., Fan X., Ma R., Wang S., Li L. (2021). Network pharmacology integrated molecular docking reveals the bioactive components and potential targets of Morinda officinalis–Lycium barbarum coupled-herbs against oligoasthenozoospermia. Sci. Rep..

[39] Xiao Z., Liu C., Duan J., Zhou T., Liu X., Lu S., Xu F. (2020). A Network Pharmacology Approach to Investigate the Anti-Depressive Mechanism of Gardeniae fructus. Int. J. Pharmacol..

[40] Yan H.-Y., Zou C.-C. (2022). Study on anticoagulant material basis and mechanism of Trichosanthis Semen and its shell and kernel based on spectrum-effect relationship integrated molecular docking. Chin. J. Chin. Mater. Med..

[41] Vidya N., Vadivukkarasi B., Manivannan G., Anbarasu K. (2008). Molecular modeling and docking studies of glutamate racemase in Vibrio vulnificus CMCP6. In Silico Biol..

[42] Cao J.-Y., Dong Q., Wang Z.-Y., Zhao Y., Ren Y., Liu C., Dang J., Yu R.-T., Tao Y.-D. (2022). Arylnaphthalide lignans from Saussurea medusa and their anti-inflammatory activities. Arab. J. Chem..

[43] Jiménez J., Doerr S., Martínez-Rosell G., Rose A.S., De Fabritiis G. (2017). DeepSite: Protein-binding site predictor using 3D-convolutional neural networks. Bioinformatics.

[44] Wang Y. (2020). Study on Mechanism of Five flavonoids Inhibiting hIAPP Aggregation and NtMGAM Activity Based on Molecular Simulation. Master’s Thesis.

[45] Zimeng A., Qianfang F., Yaruo X., Xinming Y., Yuning G., Xilian Z. (2023). Based on network pharmacology, molecular docking, and molecular dynamics simulation to explore the target mechanism of Rongchang Capsules in the treatment of pediatric epilepsy. Tianjin J. Tradit. Chin. Med..

[46] Valdés-Tresanco M.S., Valdés-Tresanco M.E., Valiente P.A., Moreno E. (2021). gmx_MMPBSA: A New Tool to Perform End-State Free Energy Calculations with GROMACS. J. Chem. Theory Comput..

[47] Pantsar T., Poso A. (2018). Binding Affinity via Docking: Fact and Fiction. Molecules.

[48] Saranya P., Karunya R., Keerthi Varshini G., Kowsikan K., Prathiksha R. (2023). In-silico docking studies of selected phytochemicals against papain like protease of SARS-CoV-2. Vegetos.

[49] Wilson Alphonse C.R., Kannan R.R. (2023). In silico exploration of antioxidants as oxidation protectant for PITRM1 peptidase activity, an Alzheimer disease target. J. Cell. Biochem..

[50] Zhao Q., Deng P.-Y., Wang H.-Y., Luo Q., Tian Y.-F., Wang G.-X., Yang Y.-Z., Li C.-G., Chen G. (2022). Molecular dynamics simulation of the interactions between puerarin and acetylcholinesterase. J. At. Mol. Phys..

[51] Ralph J.A., Morand E.F. (2008). MAPK phosphatases as novel targets for rheumatoid arthritis. Expert Opin. Ther. Targets.

[52] Liu S., Ma H., Zhang H., Deng C., Xin P. (2021). Recent advances on signaling pathways and their inhibitors in rheumatoid arthritis. Clin. Immunol..

[53] Ponce C., Torres M., Galleguillos C., Sovino H., Boric M.A., Fuentes A., Johnson M.C. (2009). Nuclear factor κB pathway and interleukin-6 are affected in eutopic endometrium of women with endometriosis. Reproduction.

[54] Liang Y., Zhou Y., Shen P. (2004). NF-kappaB and its regulation on the immune system. Cell. Mol. Immunol..

[55] Kim E.K., Kwon J.-E., Lee S.-Y., Lee E.-J., Kim D.S., Moon S.-J., Lee J., Kwok S.-K., Park S.-H., Cho M.-L. (2018). IL-17-mediated mitochondrial dysfunction impairs apoptosis in rheumatoid arthritis synovial fibroblasts through activation of autophagy. Cell Death Dis..

[56] Van Den Berg W.B., Miossec P. (2009). IL-17 as a future therapeutic target for rheumatoid arthritis. Nat. Rev. Rheumatol..

[57] Kunwar S., Dahal K., Sharma S. (2016). Anti-IL-17 therapy in treatment of rheumatoid arthritis: A systematic literature review and meta-analysis of randomized controlled trials. Rheumatol. Int..

[58] Chen H., Wang Y. (2016). Function and regulation of the osteoclast in the pathological changes of bone destruction in rheumatoid arthritis. Chin. J. Osteoporos..

[59] Perricone C., Ceccarelli F., Matteo S., Di Carlo G., Bogdanos D.P., Lucchetti R., Pilloni A., Valesini G., Polimeni A., Conti F. (2019). Porphyromonas gingivalis and rheumatoid arthritis. Curr. Opin. Rheumatol..

[60] Volin M.V. (2005). Soluble adhesion molecules in the pathogenesis of rheumatoid arthritis. Curr. Pharm. Des..

[61] Hao J., Qi F., Wang H., Su L., Li X., Zhang N., Sun W., Wei W. (2022). Network pharmacology-based prediction of inhibiting leukocyte recruitment and angiogenesis of total glucosides of peony against rheumatoid arthritis. Ann. Palliat. Med..

[62] Tang M., Zeng Y., Peng W., Xie X., Yang Y., Ji B., Li F. (2022). Pharmacological aspects of natural quercetin in rheumatoid arthritis. Drug Des. Dev. Ther..

[63] Liu X., Tao T., Yao H., Zheng H., Wang F., Gao Y. (2023). Mechanism of action of quercetin in rheumatoid arthritis models: Meta-analysis and systematic review of animal studies. Inflammopharmacology.

[64] Hou Y., Wu J., Huang Q., Guo L. (2009). Luteolin inhibits proliferation and affects the function of stimulated rat synovial fibroblasts. Cell Biol. Int..

[65] Choi E.M., Lee Y.S. (2010). Luteolin suppresses IL-1β-induced cytokines and MMPs production via p38 MAPK, JNK, NF-kappaB and AP-1 activation in human synovial sarcoma cell line, SW982. Food Chem. Toxicol..

[66] Chen W.P., Yang Z.G., Hu P.F., Bao J.P., Wu L.D. (2015). Acacetin inhibits expression of matrix metalloproteinases via a MAPK-dependent mechanism in fibroblast-like synoviocytes. J. Cell. Mol. Med..

[67] Tanigawa N., Hagiwara M., Tada H., Komatsu T., Sugiura S., Kobayashi K., Kato Y., Ishida N., Nishida K., Ninomiya M. (2013). Acacetin inhibits expression of E-selectin on endothelial cells through regulation of the MAP kinase signaling pathway and activation of NF-κB. Immunopharmacol. Immunotoxicol..

